# Supplementary material for: Model-driven characterization of functional diversity of Pseudomonas aeruginosa clinical isolates with broadly representative phenotypes
Source: Microb Genom. 2024 Jun 5;10(6):001259. doi: 10.1099/mgen.0.001259 (PMC11261902; doi:10.1099/mgen.0.001259)
Supplement: Uncited Fig. S1. [file mgen-10-01259-s001.pdf]

Supplemental figures

Model-driven characterization of functional diversity of *Pseudomonas aeruginosa* clinical isolates with broadly representative phenotypes

Mohammad Mazharul Islam, Glynis L. Kolling, Emma M. Glass, Joanna B. Goldberg, and Jason A. Papin

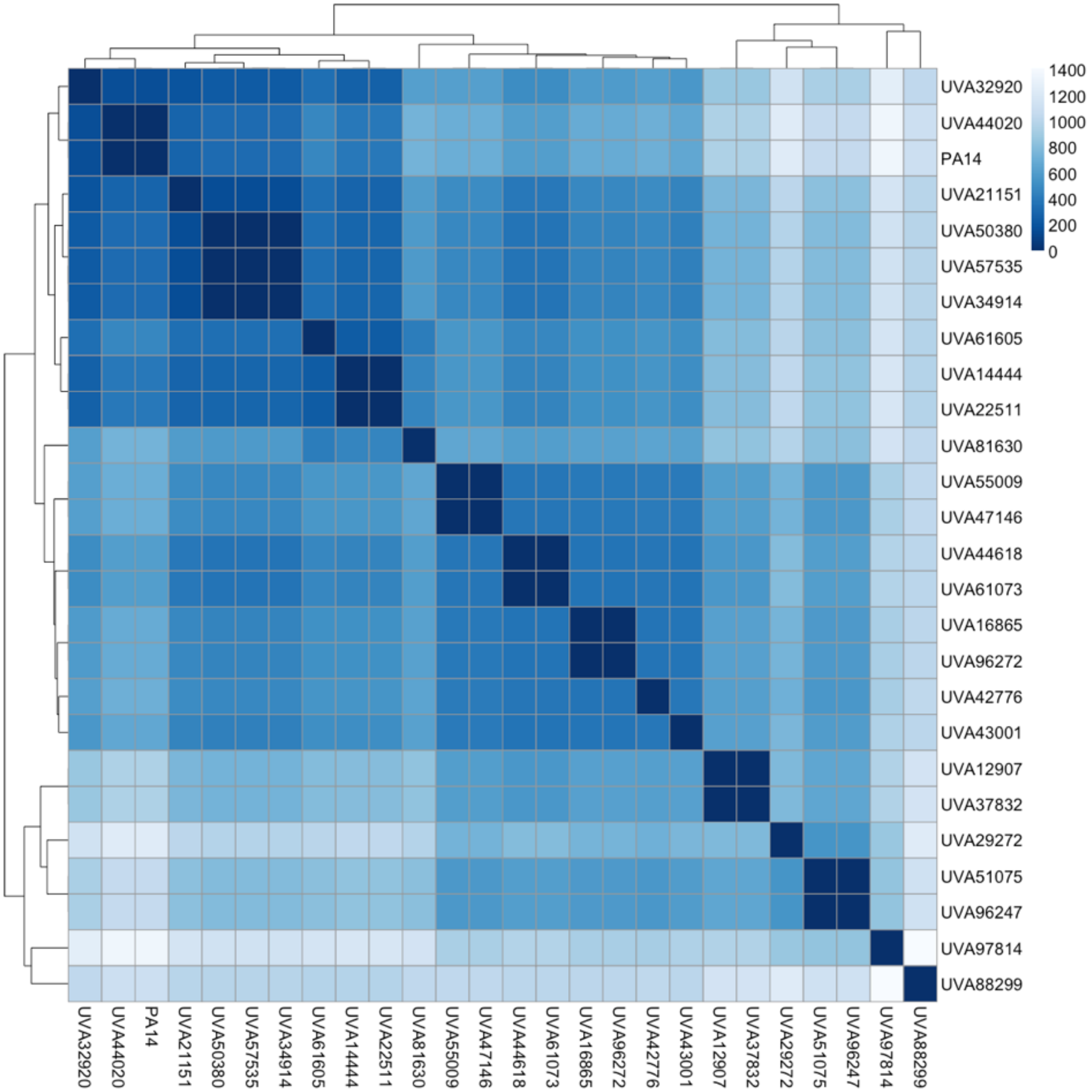

Supplemental Figure 1: Clustering of 25 clinical *P. aeruginosa* isolates based on core-genome MLST (cgMLST) profiles.

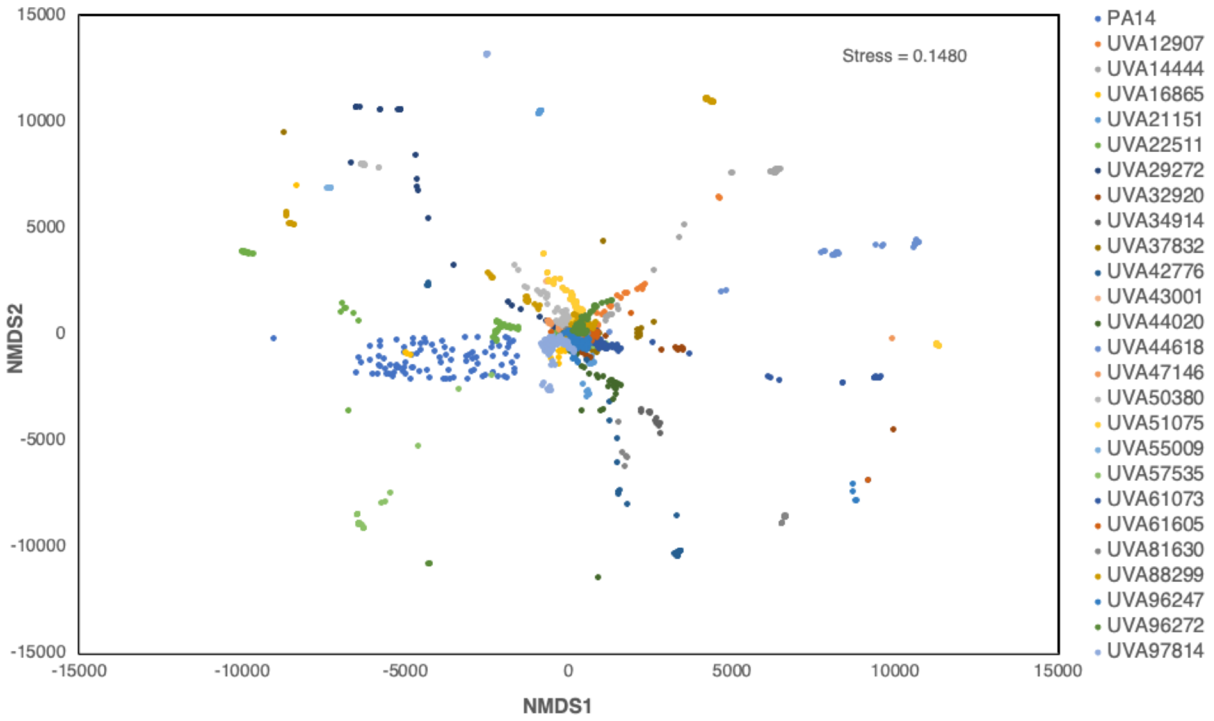

Supplemental Figure 2: NMDS plot of flux sampling data from 25 isolate metabolic reconstructions.

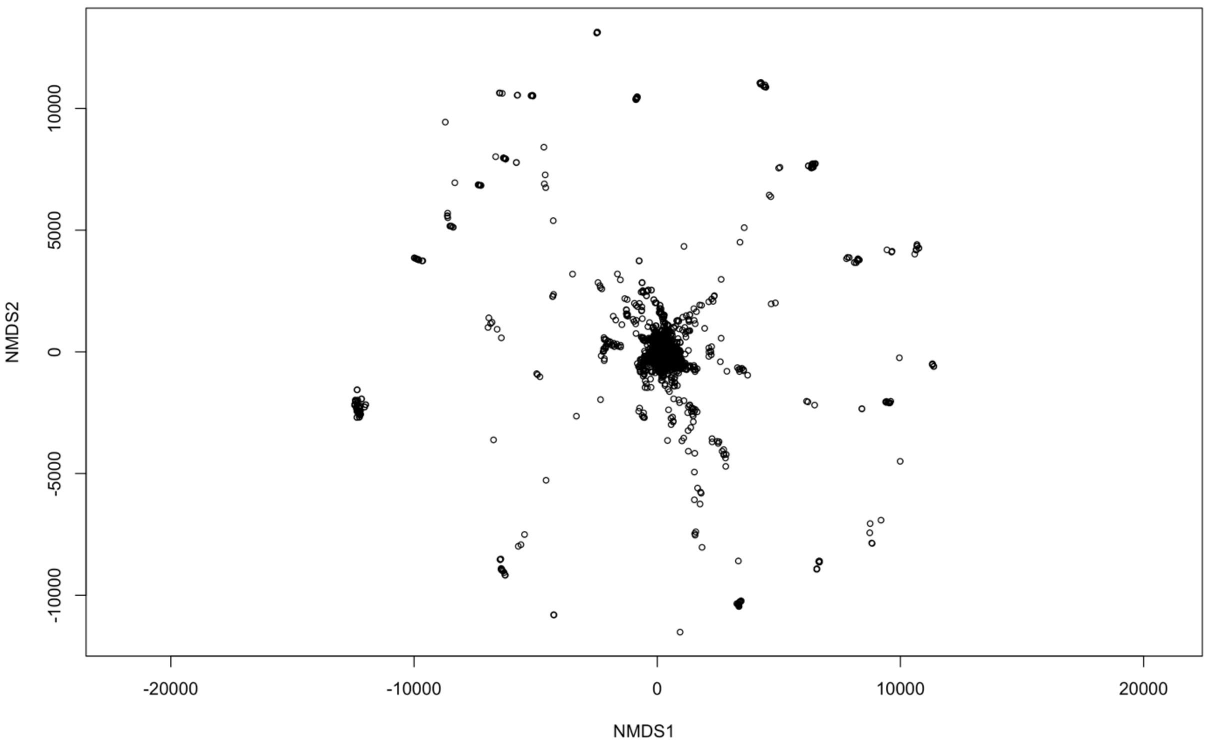

Supplemental Figure 3: NMDS plot of flux sampling data from 25 isolate metabolic reconstructions using 10,000 samples per isolate.

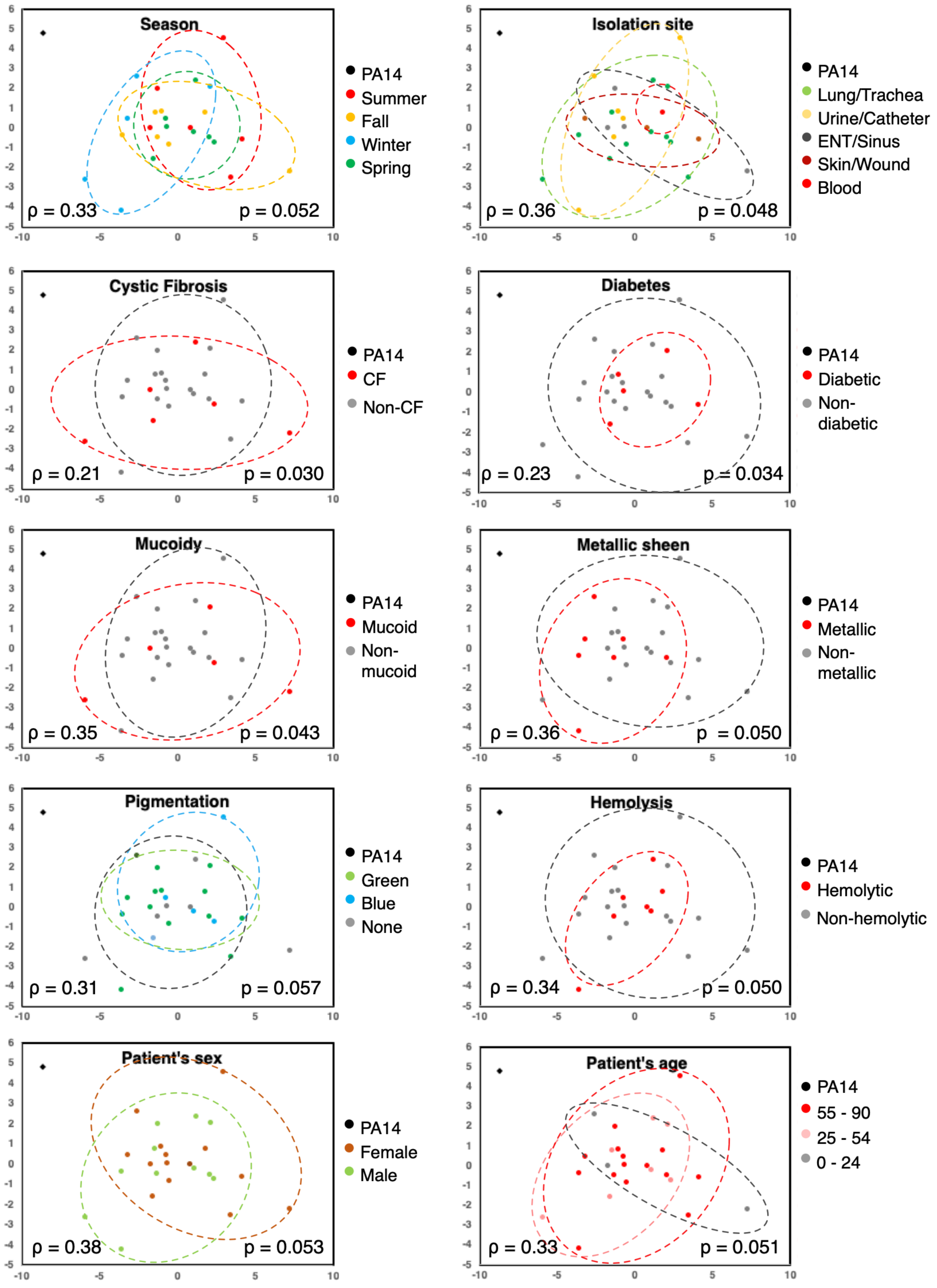

Supplemental Figure 4: NMDS plots of functional content of the 25 selected isolates and colored according to phenotypic data.
